# Genome Sequence of Novel Cluster GF Bacteriophage Wrackline, Isolated on *Microbacterium sp.* Casco Bay

**DOI:** 10.17912/micropub.biology.002002

**Published:** 2026-09-17

**Authors:** Kit Williams, Emily Savage

**Affiliations:** 1 Biology, Southern Maine Community College, South Portland, ME, United States

## Abstract

Wrackline is a novel bacteriophage isolated from sand collected along the high tide line and beneath organic material on Willard Beach, ME. It was isolated using *Microbacterium sp.* strain Casco Bay, a marine bacterium, and is also capable of infecting *Microbacterium foliorum,* a bacterium originally isolated from grass. Wrackline's genome is 42,148 bp and encodes 80 putative protein coding genes, 26 of which have no known homologs. Based on gene content similarity, Wrackline is assigned to actinobacteriophage cluster GF.

**Figure 1. Plaques, TEM image, and host range assessment of Bacteriophage Wrackline:** **Figure 1: A:** Bacteriophage Wrackline forms clear plaques approximately 0.4 – 0.6mm in diameter (n=5). **B:** Transmission electron micrograph of bacteriophage Wrackline displaying a siphovirus morphology (Hitachi HT7800, 120kV, accelerating voltage 100kV) using negative stain (1% uranyl acetate). Wrackline has an icosahedral capsid 60nm in diameter and a tail of 150nm in length (n=1). Scale bar = 200 nm. **C:** Spot dilution assay of phage Wrackline and Cluster EE phage Gardevoir ( + control) plated with *M. foliorum* plated on PYCa, and *Microbacterium sp. Casco Bay* on Marine agar *.* Dilutions 10 ^-5^ through 10 ^-7^ show Wrackline shows similar plating efficiency on both bacterial hosts.

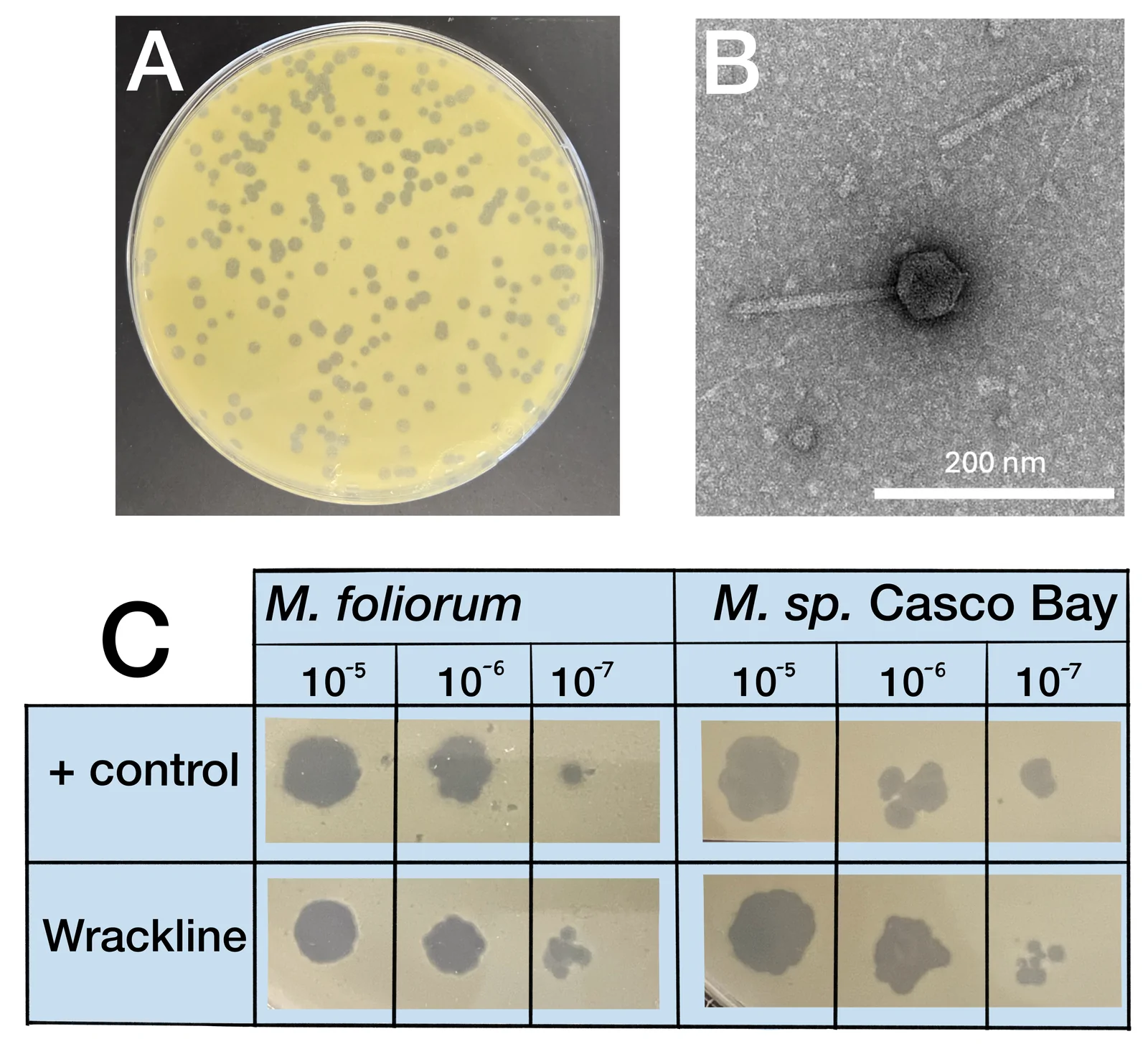



## Description

Gram-positive marine bacteria are understudied and typically challenging to culture (Gontang et al., 2007; Watkins 2022). *Microbacterium sp.* Casco Bay is halotolerant and simple to culture, making it an ideal host for the isolation and characterization of marine bacteriophage (Girard et al., 2025). While the whole genome of *M. sp.* Casco Bay has not yet been sequenced, 16S rRNA gene sequencing categorized it as a microbacterial strain.

Bacteriophage Wrackline was isolated from a sand sample taken just above the high tide line on Willard Beach, South Portland, Maine (Global Positioning System [GPS] 43.645167 N, -70.227596 W). The phage was isolated using standard procedures (Zorawik et al., 2024). Marine broth (Table 1) inoculated with *M. sp.* Casco Bay and five grams of sand were combined and incubated for 4 days at 30°C. The culture was filtered (0.22 μm pore size) and the filtrate spotted on to solidified marine top agar (Table 1) supplemented with *M. sp.* Casco Bay on a plate of marine agar (MA) (Table 1). After 48 hours at 30°C, Wrackline formed plaques with a clear center approximately 0.4–0.6mm wide (n=5) (Fig 1. A). Negative stain transmission electron microscopy showed siphovirus morphology with capsid size of 60nm and a 150nm long tail (n=1) (Fig 1. B).

DNA was extracted from a Wrackline lysate using the Promega Wizard DNA clean-up kit. Sequencing library preparation was done using the NEB Ultra II Library Kit, and the library sequenced on the Illumina NextSeq 1000 sequencer with the XLEAP-PI kit. This yielded 2.6 million 100 base single-end reads, providing 5,885-fold coverage. Raw reads were trimmed and filtered by cutadapt 4.7 using the -nextseq-trim 30 argument (Martin 2011), and skewer 0.2.2 using options -q 20 -Q 30 -n -l 50 (Jiang et al., 2014) and genomic termini and completeness was checked using Consed V29 (Gordon and Green, 2013) default parameters. Wrackline's genome is 42,148 bp with a 69.2% GC content and is circularly permuted. 

Wrackline was automatically annotated in DNA Master v5.23.6, build 2705 (Pope and Jacobs-Sera, 2017) using Glimmer (v3.02b) (Delcher et al., 2007) and GeneMark (v2.5p) (Besemer and Borodovsky, 2005) to assess coding potential and identify putative genes. Start sites were then refined and gene functions predicted using Phamerator (Actino_Draft v597) (Cresawn et al., 2011), Starterator (v3.02) (Pacey, 2016), HHPred (v2.08 (PDB_mmCIF70, SCOPe70, Pfam-A, NCBI_Conserved_Domains [CD]) (Söding et al., 2005), and Blastp (Actinobacteriophage database, NCBI non-redundant database) (Altschul et al., 1990). DeepTMHMM (v1.2.33.c.) (Hallgren et al., 2022) and SOSUI (v1.11) (Hirokawa et al., 1998) were used to assess the presence of transmembrane proteins. No tRNA genes were detected using Aragorn (tRNA-scan SE v1.2.41 v). Default parameters were used for all software programs. 

This analysis identified 80 predicted protein-coding genes, 26 of which are orphams with no known homologs (Cresawn et al., 2011). The left arm of the genome is transcribed in the forward orientation and comprised of structural genes and the endolysin, followed by a reverse transcribed region containing genes of unknown functions and DNA binding domains, then returning to the forward orientation. The right arm of the genome contains a RecE-like exonuclease and RecT-like DNA pairing protein (GP 64 and 65) which may work together as a recombinase. 

Cluster assignment based on gene content similarity (GCS) of at least 35% to other phages in the Actinobacteria database (Actino_Draft database) placed Wrackline in cluster GF which only contains 3 other phages to date (Pope et al., 2017; Russell and Hatfull, 2017). The other cluster GF phages share more than 76.3% GCS, while Wrackline stands out sharing only 48.6% GCS with the group. While the structural genes of Wrackline are similar to other GF phages, the first 12 genes vary from others in the cluster and among those are 8 orphams for which no function was assigned. The reverse section of the genome from GP 38 – 60 shares some protein phams with other GFs, but is an orpham rich region, with 7 orphams with no functional assignment. Additionally, other phages in the cluster were isolated using *Microbacterium foliorum.* Host range for phage Wrackline was evaluated by standard procedures (Zorawik et al., 2024). Spot plates were prepared using Wrackline and a control phage (Gardevoir (EE)) known to infect *Microbacterium foliorum* and *Microbacterium sp.* Casco Bay. Phage lysate for Wrackline and Gardevoir were diluted to 10 ^-7^ and2uL were spotted onto each host. Wrackline is able to infect *M. foliorum* with a similar plating efficiency (Fig 1. C).

**Nucleotide sequence accession numbers**

Wrackline is available at GenBank with Accession No. PV876983 and Sequence Read Archive (SRA) No. SRX29714291 . The host strain Microbacterium sp. Casco Bay is archived in the National Center for Marine Algae and Microbiota (NCMA) at Bigelow Laboratory for Ocean Science (B81).

## Reagents

**Table:** 

Marine Agar Recipies for M. sp. Casco Bay	
Marine Broth	750mL filtered seawater
	250mL distilled water
	0.5g yeast extract
	0.5g casamino acids
	3mL glycerine
Marine Agar	750mL filtered seawater
	250mL distilled water
	1g yeast extract
	5g bacto peptone
	15g agar
Marine Top Agar	750mL filtered seawater
	250mL distilled water
	0.5g yeast extract
	0.5g bacto peptone
	0.5g cas amino acids
	5g agar
	1mL of 1M CaCL2 per 100mL is added post-autoclave
PYCa Recipies for M. foliorum	
PYCa Broth	1L distilled water
	1g yeast extract
	15g bacto peptone
	2.5mL 40% dextrose
PYCa Agar	1L distilled water
	1g yeast extract
	15g bacto peptone
	2.5mL 40% dextrose
	15g agar
	1mL cycloheximide (10mg/mL) added post-autoclave
PYCa Top Agar	1L distilled water
	1g yeast extract
	15g bacto peptone
	2.5mL 40% dextrose
	7g agar
	1mL cycloheximide (10mg/mL) added post-autoclave
	1mL of 1M CaCL2 per 100mL is added post-autoclave
